# Cognitive Load Affects Numerical and Temporal Judgments in Distinct Ways

**DOI:** 10.3389/fpsyg.2018.01783

**Published:** 2018-10-02

**Authors:** Karina Hamamouche, Maura Keefe, Kerry E. Jordan, Sara Cordes

**Affiliations:** ^1^Boston College, Chestnut Hill, MA, United States; ^2^Department of Psychology, Utah State University, Logan, UT, United States

**Keywords:** quantity processing, time perception, number processing, cognitive load, quantity estimation

## Abstract

Prominent theories suggest that time and number are processed by a single neural locus or a common magnitude system (e.g., Meck and Church, 1983; Walsh, 2003). However, a growing body of literature has identified numerous inconsistencies between temporal and numerical processing, casting doubt on the presence of such a singular system. Findings of distinct temporal and numerical biases in the presence of emotional content (Baker et al., 2013; Young and Cordes, 2013) are particularly relevant to this debate. Specifically, emotional stimuli lead to temporal *over*estimation, yet identical stimuli result in numerical *under*estimation. In the current study, we tested adults’ temporal and numerical processing under cognitive load, a task that compromises attention. Under the premise of a common magnitude system, one would predict cognitive load to have an identical impact on temporal and numerical judgments. Inconsistent with the common magnitude account, results revealed baseline performance on the temporal and numerical task was not correlated and importantly, cognitive load resulted in distinct and opposing quantity biases: numerical underestimation and marginal temporal overestimation. Together, our data call into question the common magnitude account, while also providing support for the role of attentional processes involved in numerical underestimation.

## Introduction

Throughout our daily lives, we constantly track temporal and numerical information. However, this process is never void of context; in fact, it is often coupled with distractions. We often calculate a tip at a restaurant while simultaneously talking to our friends or estimate how long it will take us to drive home while listening to a child crying in the backseat. Although research has most frequently tested quantity processing in controlled laboratory settings, recent work has revealed quantity processing biases in the presence of external stimuli. For example, these studies have revealed durations to be *over*estimated and numerosities to be *under*estimated in the presence of emotional content, namely angry faces (see Gil et al., 2007; Baker et al., 2013; Young and Cordes, 2013). These findings have led many researchers to re-think prominent theories of quantity processing, while also unveiling questions regarding the cognitive mechanism(s) involved in quantity processes. In the current study, we investigated adults’ temporal and numerical processing under cognitive load – an attention-distracting working memory task. This manipulation not only mimics real-world quantity processing, but allows us to directly test theories of quantity processing.

Evidence from behavioral, neural, and clinical data reveal many striking parallels in numerical and temporal processing (Dormal et al., 2006; Feigenson, 2007; Provasi et al., 2011; Dormal and Pesenti, 2012, 2013; Vicario et al., 2013). For example, behavioral data indicate that the ease of numerical and temporal judgments relies on Weber’s Law (Stevens, 1957). That is, it is easier to discriminate between two numerosities or durations when they differ by a larger ratio. Other behavioral work shows that rats and infants are able to generalize a rule learned in a numerical domain to a temporal domain and vice versa (Meck and Church, 1983; de Hevia et al., 2012). Neuroimaging and clinical work also reveals similarities between numerical and temporal processing. For instance, adults’ intraparietal sulcus (IPS) is activated while processing both numerical and temporal stimuli (Dormal et al., 2012; Skagerlund et al., 2016). Relatedly, Hayashi et al. (2013); Experiment (1) show comparable activations in the intraparietal cortex and inferior frontal gyrus during temporal and numerical discrimination tasks. Moreover, many individuals’ suffering from clinical disorders, such as Turner Syndrome, also experience comorbid quantity processing deficits (e.g., Vicario et al., 2013), further emphasizing commonalities in quantity processing. These findings have led researchers to propose that a single neural locus, or a common magnitude system, is responsible for processing both time and number (Meck and Church, 1983; Walsh, 2003; Cantlon et al., 2009).

Despite reports of numerous similarities in numerical and temporal processing, researchers have also identified many striking inconsistencies in processing these two types of quantity (e.g., Baker et al., 2013; Young and Cordes, 2013; Odic, 2017). Although number and time follow comparable developmental trajectories in infancy, different developmental trajectories occur in childhood (Odic, 2017). While some work has suggested that the IPS is activated during both temporal (e.g., Schubotz et al., 2000; Rao et al., 2001) and numerical (e.g., Cantlon et al., 2006; Piazza et al., 2007; Ashkenazi et al., 2008) tasks, the overall consensus is that the IPS is implicated in numerical, but not temporal, processing (Rammsayer and Classen, 1997; Nenadic et al., 2003; Mattel and Meck, 2004; Koch et al., 2009). Research revealing unique numerical and temporal biases when making quantity judgments in the presence of emotional content has been particularly damaging to the common magnitude account. In these studies, participants passively view a happy, angry, or neutrally valenced face immediately before making a numerical or temporal judgment. Results show that both children and adults consistently *underestimate* numerosity in the presence of both negatively and positively valenced stimuli (both happy and angry faces). In contrast, durations are *overestimated* in the presence of negatively valenced stimuli^1^ (angry faces; Gil et al., 2007; Baker et al., 2013; Young and Cordes, 2013). That is, the exact same emotional stimuli differentially bias numerical and temporal processing, challenging claims of a common magnitude system. In the current study, we investigate the effect of cognitive load on temporal and numerical judgments. While comparable temporal and numerical biases would provide evidence in favor of a common magnitude system, unique temporal and numerical biases under cognitive load would call this account into question.

Distinct biases in the presence of emotional content have not only challenged claims of a common magnitude system, but also led researchers to speculate about the cognitive mechanism(s) underlying numerical and temporal processing. Because distinct patterns of estimation occur in the presence of emotional faces – only angry faces lead to temporal overestimation, but both angry and happy faces lead to numerical underestimation – some have explained these results by the differential effects of arousal and attention on quantitative processing (Young and Cordes, 2013). While previous work has tested the effects of altered attention or heightened arousal on temporal and numerical judgments separately (e.g., Thomas and Brown, 1974; Rammsayer and Lima, 1991; Brown and Stubbs, 1992; Macar et al., 1994; Brown, 1997, 2008; Casini and Macar, 1997; Fortin and Rousseau, 1998; Zakay, 1998; Khan et al., 2006; Wearden et al., 2007; Block and Zakay, 2008; Ortega and Lopez, 2008; see Block et al., 2010; Hamamouche et al., 2017), these studies have rarely investigated these manipulations on temporal and numerical processing in the same individuals using the same task. Moreover, attention and arousal are closely related constructs, and are inevitably involved in both temporal and numerical estimation. Thus, in the current study, we aimed to solely manipulate attention – defined as “the appropriate allocation of processing resources to relevant stimuli” (Coull, 1998, p. 344) – by introducing cognitive load during temporal and numerical processing in the same individuals. Using this manipulation, we will be able to (1) assess the impact of attention manipulations on temporal and numerical processing, and (2) determine whether comparable biases occur in the presence of an attention distracting task.

### The Current Study

In the current study, adults made temporal and numerical judgments under cognitive load – a distracting, working memory task – in order to assess the likelihood of the common magnitude system. Under the premise of a common magnitude system, one would predict altered attention from the cognitive load manipulation to identically impact numerical and temporal judgments. However, if numerical and temporal processing are dictated by distinct cognitive systems, temporal and numerical biases under cognitive load may not track together.

## Materials and Methods

### Participants

Eighty Boston College undergraduates participated in this study for course credit or cash compensation (58 females, *M*_age_ = 19.15 years). After exclusions (see criteria below), there were 71 participants with complete data.

### Experimental Design and Procedure

Participants completed both a numerical and a temporal bisection task. Within each task, there were two blocks: a baseline block and a cognitive load block. The order of the tasks (numerical vs. temporal) and the order of the blocks within each task (baseline vs. cognitive load) were counterbalanced. However, the order remained consistent within each participant – if the participant completed the cognitive load trials first in the temporal bisection task, s/he would also complete the cognitive load trials first in the numerical bisection task.

In the numerical bisection task, participants were first familiarized to a small (15 dots) and a large (60 dots) standard value (modeled after Lewis et al., 2017). Each display was shown twice and labeled both on the screen and by the experimenter with the appropriate size (i.e., small or large). To confirm that participants knew the standard values before beginning the test trials, participants first completed four standard practice trials in which they had to classify the dot arrays containing the standard values as either small or large (by pressing a button on the keyboard). Participants received feedback on each of the four standard practice trials. No other feedback was provided during the experiment.

In the baseline block of the numerical task, participants then completed additional practice trials in which they were presented with arrays containing intermediate numerosities (19, 24, 30, 38, 48) in addition to the standard values and were asked to indicate whether the numerosity of the display was more similar to the small or large standard. Each numerosity (standard values and intermediate values) was shown once in a randomized order, resulting in seven baseline practice trials. Participants completed these baseline practice trials to familiarize them to the demands of the task. After completing the practice trials, participants then completed the baseline test trials, during which each of the seven numerosities were presented 12 times each in a random order, resulting in a total of 84 test trials. Dot arrays were presented for 750 ms. For each numerosity, there were twelve different configurations of dots. Within each array, the size of all dots was held constant; however, dot sizes varied across arrays. Half of the arrays controlled for cumulative surface area, such that regardless of the numerosity, the cumulative area of the array was held constant (approximately 133.6 cm^2^). Thus, individual dots were smaller as the number of dots in the array increased. The other half of the dot arrays controlled for dot size such that each dot, regardless of the numerosity of the array, had an area of 4.01 cm^2^. Thus, dot arrays with fewer dots also had a smaller cumulative surface area (cumulative areas ranged from 59.9 to 239.99 cm^2^).

**FIGURE 1:**
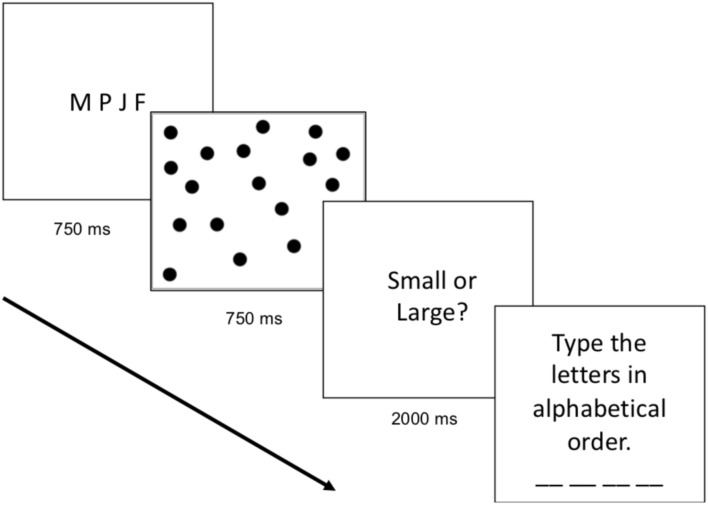
Stimulus presentation for Cognitive Load trials during the Numerical Bisection Task.

The cognitive load block was identical to the baseline block except that participants were required to remember and alphabetize four letters while simultaneously completing the numerical task. On every trial, participants saw four letters on the screen (e.g., M K F J) for 750 ms and were told to remember and alphabetize the letters. Participants were then presented with the dot array. After making their numerical judgment (whether the array was more similar to small or large standard), participants were shown a text box in which they were instructed to type the four letters in alphabetical order (e.g., F J K M). The participants first completed seven practice trials (one with each numerosity), and then completed 84 test trials (12 displays of each numerosity). During test, participants were only asked to type the letters alphabetically on a random two-thirds of the trials; however, participants did not know when they would be required to type the letters in alphabetical order, thus they were required to perform the working memory task on every trial in anticipation of receiving the prompt. The progression of the cognitive load trials can be found in **Figure 1**.

The temporal bisection task was identical to the numerical bisection, except participants saw a blue oval in the center of the screen for a specified duration instead of a dot array. Participants were familiarized to two standard durations, a short standard (400 ms) and a long duration (1600 ms; modeled after Young and Cordes, 2013). Again, participants began with four standard practice trials during which they classified the two standard durations and received trial-by-trial feedback. Participants then completed seven additional practice trials with each standard value and the intermediate durations (504, 635, 800, 1008, and 1270 ms) intermixed, during which they were instructed to decide whether the oval’s duration was more similar to the short standard or the long standard. Then during the baseline block, participants completed 84 test trials (7 durations × 12 presentations = 84 test trials). The cognitive load block was identical – participants were again shown four letters prior to every temporal stimulus and were asked to remember and alphabetize them during the temporal task as described above.

### Data Coding

#### Exclusion Criteria

Five participants only completed one of the two bisection tasks (numerical or temporal) and an additional three participants only completed one trial type (baseline or cognitive load). Thus, performance on the task or block that was not completed was coded as missing data.

Data from participants who performed poorly on test trials involving the standard values (<75% accuracy on the standard values combined) were excluded (Number: *N*_Baseline_ = 1, *N*_CognitiveLoad_ = 2; Time: *N*_Baseline_ = 1; *N*_CognitiveLoad_ = 2).

##### Cognitive load

To assess participants’ accuracy on the cognitive load task, we calculated the percentage of trials during which each participant correctly alphabetized the letters for the numerical and temporal bisection separately. Participants who typed in the letters without alphabetizing them were removed from the cognitive load analyses (*N*_Number_ = 2, *N*_Time_ = 5).

##### Bisection task dependent measures

Two dependent measures were taken from the bisection task:

(1) **Relative Point of Subjective Equality (PSE)**. The PSE corresponds to the value at which 50% of the responses were classified as “large” (or “long”). First, the proportion of responses during which the participant judged the numerosities (or durations) as being closer to the large (long) standard was plotted as a function of the stimulus numerosity (or duration) and these data were fit with a Cumulative Gaussian function (as per Çoşkun et al., 2015). These curves were then used to determine each participant’s PSE, or the value at which 50% of responses were “large” (or “long”). PSEs were calculated separately for each bisection task (numerical and temporal) and block type (baseline and cognitive load). It should be noted that a higher PSE is indicative of a leftward shift in the curve, indicative of a lower likelihood of the participant judging the value as similar to the long/large standard; i.e., it is indicative of underestimation. PSE values that were three standard deviations above or below the mean were replaced with the next highest/lowest value within that range (Time: *N*_Baseline_ = 1, *N*_CognitiveLoad_ = 1). Because PSE varies based on the range of values for each task (15–60 for number and 400–1600 for time), we calculated a Relative PSE for each task and trial block separately. The Relative PSE was calculated by dividing each participant’s PSE by the geometric mean of the standard values (30 for the numerical task and 800 for the temporal task), thus allowing for direct comparisons between temporal and numerical performance.
(2) **Relative Difference Limen (DL)**. The DL is a measure of the participant’s consistency in responding and corresponds to the value halfway between the set sizes corresponding to a 75% probability of a large/long response and a 25% probability of a large/long response. Outliers were replaced with the next largest/smallest value within the range (Time: *N*_Baseline_ = 1, *N*_CognitiveLoad_ = 1; Number: *N*_Baseline_ = 1). Again, we calculated the Relative DL by dividing each participants’ DL by the geometric mean of the standard values.

## Results

First, we confirmed that the order in which participants completed the bisection task (numerical versus temporal first) and/or the block order (baseline trials versus cognitive load trials first) did not interact with our variables of interest (Relative PSE, Relative DL). Neither the order in which participants completed the bisection tasks (numerical versus temporal first) nor the order of the blocks (baseline vs. cognitive load) interacted with our variables of interest (*p*’s > 0.05); thus, we collapsed data across these variables in the subsequent analyses.

### The Relation Between Time and Number

The common magnitude account would predict a correlation between baseline performance on the numerical and temporal bisection. However, performance on the baseline numerical and temporal tasks was not correlated (Relative PSE: *r* = 0.090, *p* = 0.445, Relative DL: *r* = 0.132, *p* = 0.263).

### Cognitive Load Performance

Next, we tested whether cognitive load accuracy (i.e., correctly alphabetizing the four letters) differed as a function of the task (numerical versus temporal bisection). This was done to ensure that cognitive load affected participants in each task approximately equally. A paired samples *t*-test revealed no significant difference in cognitive load accuracy across the two tasks, *t*(72) = 0.723, *p* = 0.472 (Temporal Task: *M* = 0.70, *SE* = 0.02; Numerical Task: *M* = 0.69, *SE* = 0.02).

### Effect of Cognitive Load on Temporal and Numerical Judgments

#### Relative PSE

**FIGURE 2:**
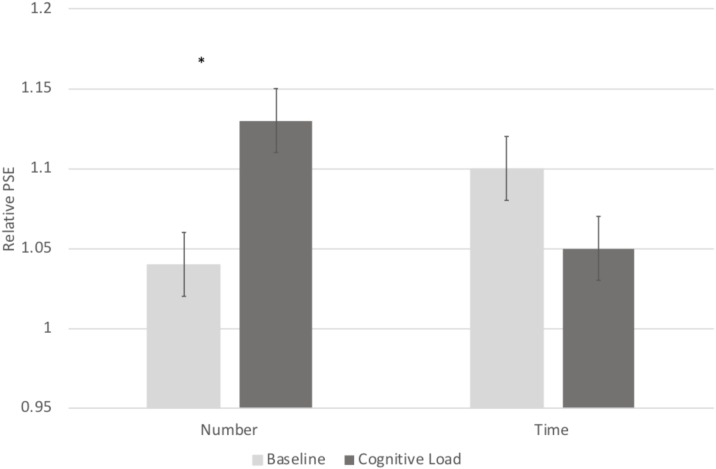
A significant Block × Trial Type interaction revealed differential effects of cognitive load on temporal and numerical judgments.

**FIGURE 3:**
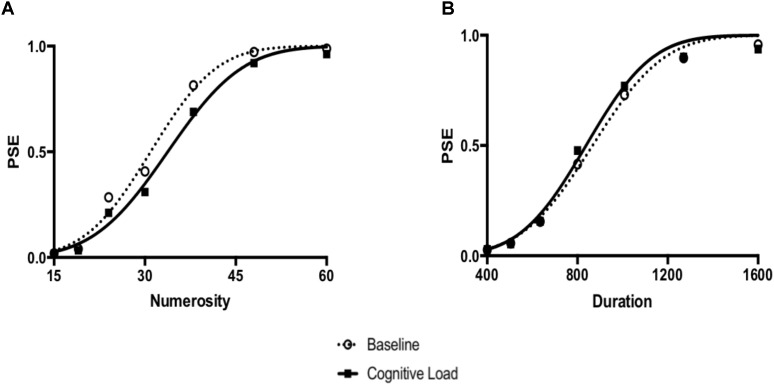
**(A)** Numerical bisection data and the best fitting Cumulative Gaussian functions using the group average. **(B)** Temporal bisection data and best fitting Cumulative Gaussian functions using the group average.

**FIGURE 4:**
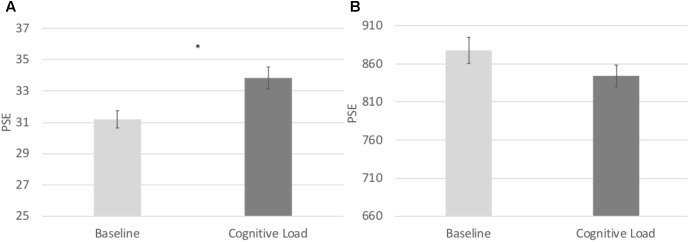
**(A)** Point of subjective equality estimates from the Numerical Bisection Task. **(B)** Point of subjective equality estimates from the Temporal Bisection Task.

In order to directly compare biases on the numerical and temporal task, we conducted a 2 (Task: numerical vs. temporal bisection) × 2 (Block: baseline versus cognitive load) repeated measures ANOVA on the Relative PSE. There was a significant Task × Block interaction, *F*(1, 71) = 22.063, *p* < 0.001, $\eta_{p}^{2}$ = 0.237 (See **Figure 2**). No other main effects reached significance, *p*’s > 0.2. To follow up on the Task × Block interaction, we next conducted paired samples *t*-tests comparing the baseline and cognitive load trials within the numerical and temporal task separately. Cognitive load trials (*M* = 1.13, *SE* = 0.02) were significantly underestimated compared to baseline trials (*M* = 1.04, *SE* = 0.02) in the numerical bisection, *t*(75) = -4.634, *p* < 0.001, See **Figures 3A, 4A** whereas cognitive load trials (*M* = 1.05, *SE* = 0.02) were marginally overestimated compared to baseline trials (*M* = 1.10, *SE* = 0.02) in the temporal bisection, *t*(75) = 1.890, *p* = 0.063, See **Figures 3B, 4B** providing support for the role of attention in numerical processing by indicating numerical underestimation under cognitive load. Lastly, we conducted additional paired samples *t*-tests to compare numerical and temporal processing in each block separately. Performance at baseline was comparable on the numerical and temporal task during the baseline trials *t*(73) = -1.990, *p* = 0.05. However, under cognitive load performance, the relative PSE on the numerical task was significantly greater than the temporal, *t*(71) = 2.743, *p* = 0.008, emphasizing the unique effect of cognitive load on the two bisection tasks.

#### Relative DL

**FIGURE 5:**
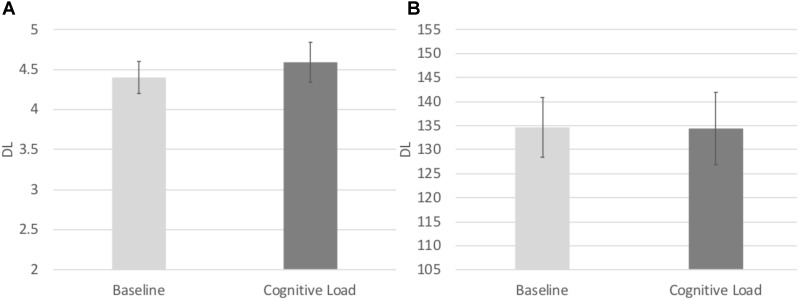
**(A)** Difference limen estimates from the Numerical Bisection Task. **(B)** Difference limen estimates for the Temporal Bisection Task.

In order to test the effects of cognitive load on the consistency of participants’ responding, we conducted identical repeated measures ANOVA on the Relative DL in both the numerical and temporal tasks. There was a main effect of task, *F*(1, 71) = 4.841, *p* = 0.031, $\eta_{p}^{2}$ = 0.064, such that the Relative DL on the numerical task (*M* = 0.147, *SE* = 0.006) was significantly lower to that of the temporal task (*M* = 0.165, *SE* = 0.007), indicative of more consistent responding in the numerical task across both Blocks. No other main effects or interactions reached significance, *p*’s > 0.8 (see **Figure 5**).

## Discussion

Understanding how quantity processing occurs in the real world is critical for assessing prominent theories of quantity processing and can also shed light on the cognitive mechanism(s) underlying these processes. Previous research identified numerous similarities in processing quantities such as time and number, leading to the prominent common magnitude system theory (see Walsh, 2003). However, newer work revealing many discrepancies in quantity processing (e.g., Baker et al., 2013; Odic, 2017) has undercut the common magnitude account. Of particular interest to this line of work are findings that angry face stimuli lead to numerical underestimation, yet temporal overestimation. These results have led researchers to rethink the prominent theory of a common magnitude system and consider the alternative that distinct cognitive mechanism(s) may underlie numerical and temporal processing. In the current study, we used a cognitive load manipulation to directly test the effect of altered attention during temporal and numerical processing in adults.

### Temporal and Numerical Processing in Baseline Conditions

First, the common magnitude system would predict performance on comparable temporal and numerical tasks to be correlated. Replicating previous work, our baseline data revealed no correlation between performance on our temporal and numerical tasks (see Agrillo et al., 2013; Young and Cordes, 2013; Odic et al., 2016). This finding matches several null results from other research groups, further undercutting the common magnitude account by suggesting that one’s ability to track time and number are dictated by distinct patterns of representational acuity even within the same individuals.

### Quantity Biases Under Cognitive Load

The common magnitude system would not only predict a correlation between temporal and numerical processing, but also temporal and numerical biases to track in the same direction under identical conditions. Despite this, several studies have demonstrated unique temporal and numerical biases in the presence of emotional content (Gil et al., 2007; Baker et al., 2013; Young and Cordes, 2013; Lewis et al., 2017). While these biases have previously been observed in the presence of emotional content, our data provide additional evidence for differential biases in temporal and numerical processing in a new context – during cognitive load. Mirroring earlier work with emotional content, our data reveal that cognitive load led to numerical underestimation. Temporal judgments, however, were marginally overestimated during cognitive load trials. Our findings join others challenging claims of the common magnitude system by identifying distinct and opposing biases in temporal and numerical processing (Gil et al., 2007; Baker et al., 2013; Young and Cordes, 2013; Lewis et al., 2017).

Our study also aimed to further explore the effect of attention on temporal and numerical processing. While previous work has suggested heightened arousal leads to temporal overestimation, but altered attention leads to numerical underestimation (Young and Cordes, 2013), attention and arousal are related constructs. Thus, our study attempted to solely test the effect of altered attention – through the induction of cognitive load – on both temporal and numerical processing within the same individuals. As predicted, numerical judgments were underestimated during the critical cognitive load trials, suggesting that numerical processing was disrupted while concurrently performing a distracting working memory task.

It is also important to note that temporal judgments were also marginally impacted by cognitive load, but in a different direction and less robustly. Unlike the findings with the numerical data, cognitive load resulted in the marginal overestimation of durations. These findings somewhat replicate previous work demonstrating that individuals tend to overestimate time in the presence of angry stimuli (e.g., Gil et al., 2007; Young and Cordes, 2013). However, they are counter to findings suggesting that altered attention leads to shorter duration estimates (e.g., Brown and Stubbs, 1992; Brown, 1997, 2008; Block and Zakay, 2008; Block et al., 2010). The opposing findings of our study compared to previous work may be accounted for by methodological differences, such as the type of temporal task employed and the durations used. Although prior work has primarily explored attentional manipulations on timing in the context of production or reproduction tasks (which typically assess estimation), the current study employed a bisection task that assesses subjective temporal judgments. In line with this possibility is work revealing differential impacts of *arousing* stimuli on temporal judgments across different timing tasks. Although angry faces lead to temporal overestimation in bisection, estimation, and production tasks, emotion does not impact temporal performance on reproduction or generalization tasks (Gil and Droit-Volet, 2011). While differences in the task demands could have led to discrepancies between our study and others, research employing bisection tasks, like the one used in the current study, has also revealed temporal underestimation (see Casini and Macar, 1997; Droit-Volet et al., 2010; Tipples, 2010). Moreover, our task focused on short durations (<1600 ms), yet previous work has typically, although not exclusively, tested the effect of cognitive load on longer durations (>2 s). This is particularly important given substantial evidence for two separate timing systems for timing sub-seconds (<1 s) and supra-seconds (>1 s, e.g., Lewis and Miall, 2003; Buhusi and Cordes, 2011; Gooch et al., 2011). Thus, it is possible that either the task employed, or the specific durations tested, may have led our findings to conflict with previous work on timing and attention. Regardless, our data further emphasize the need to investigate the effect of cognitive load on both sub- and supra-second judgments across tasks to determine whether how attentional manipulations impact timing judgments.

Lastly, because cognitive load was expected to impact attention, it was predicted that the inclusion of a dual task paradigm would have led to less consistent responding on the cognitive load trials. Surprisingly, this was not the case. Our data analyses revealed that introducing cognitive load did not impact participants’ consistency in making temporal or numerical judgments. In fact, our only finding in regards to response consistency was an overall main effect of task, such that numerical judgments were more precise than temporal judgments. This finding is consistent with evidence indicating that numerical judgments are more accurate than temporal judgments across the lifespan (Droit-Volet et al., 2008, Expt 1; Odic et al., 2016; Odic, 2017).

While our goal was to directly manipulate attention, it is important to note that our cognitive load manipulation not only altered attention, but also necessarily engaged other domain-general abilities such as working memory. Although we intentionally chose a cognitive load manipulation that has been used in the literature for manipulating attention (e.g., Postle et al., 1999), it is clear that this secondary task confounded attention and working memory. Thus, it is possible that the biases obtained in our data may be the result of manipulations to another domain general cognitive process, rather than attention specifically. Future work will be important for teasing apart this alternative. In particular, studies employing eyetracking methods, in which implicit measures of attention could be directly measured, would be particularly beneficial for investigating this possibility.

## Conclusion

These data provide evidence against a common magnitude system by (a) demonstrating inconsistencies in representing different types of quantity within individuals, and (b) showing numerical underestimation, but slight temporal overestimation during an attention distracting task. Although our findings suggest that attention is critical for numerical processing, more work is needed to shed light on *how* attentional manipulations impact quantity processing. Future research will be critical for understanding the unique representational formats of quantities, and additional work exploring the role of attention and arousal as cognitive mechanisms underlying quantity processing may be particularly fruitful for shedding light on the representational patterns of quantitative information.

## Ethics Statement

This study was carried out in accordance with the recommendations of Institutional Review Board, Boston College. The protocol was approved by the Institutional Review Board, Boston College. All subjects gave written informed consent in accordance with the Declaration of Helsinki.

## Author Contributions

KH and MK completed all data collection. KH wrote the manuscript. MK, KJ, and SC provided thoughtful feedback on the manuscript. All authors contributed to the design of the study and approved of this version of the manuscript.

## Conflict of Interest Statement

The authors declare that the research was conducted in the absence of any commercial or financial relationships that could be construed as a potential conflict of interest.

## Footnotes

1 While temporal overestimation typically occurs in the presence of angry faces, this effect seems to depend on the temporal measure used (see Gil and Droit-Volet, 2011; Lui et al., 2011).
